# Supplementary figures and images for: Effects of early geometric confinement on the transcriptomic profile of human cerebral organoids
Source: BMC Biotechnol. 2021 Oct 12;21:59. doi: 10.1186/s12896-021-00718-2 (PMC8507123; doi:10.1186/s12896-021-00718-2)

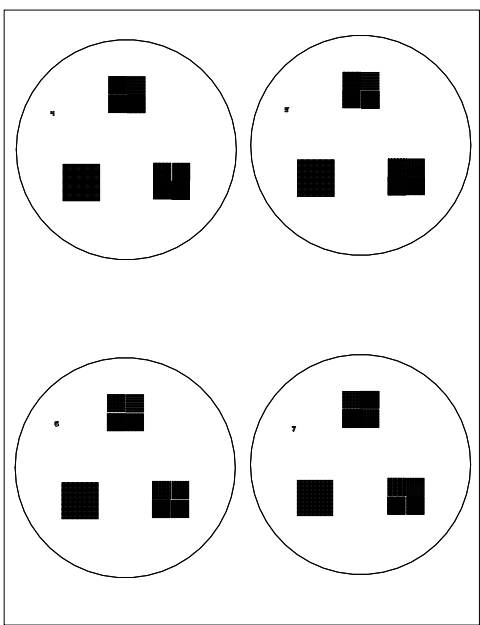

Supplement: Supplementary file 2 — Additional file 2. PDF version of AutoCAD (DWG) file related to main figure 1. Schematic files for the designs of the microwells. [file 12896_2021_718_MOESM2_ESM.pdf]

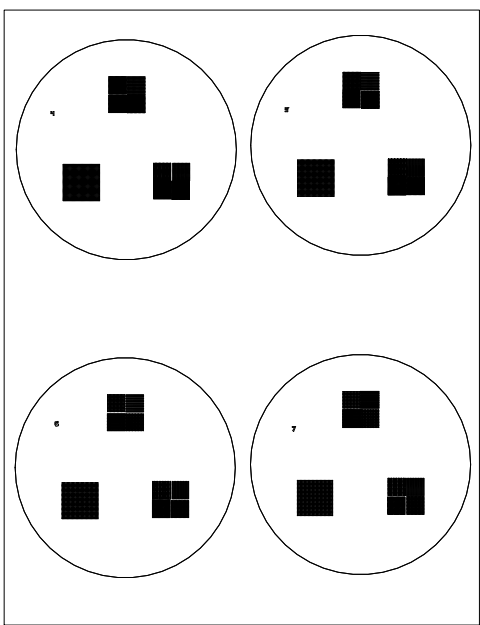

Supplement: Supplementary file 3 — Additional file 3. PDF version of AutoCAD (DXF) file related to main figure 1.Schematic files for the designs of the microwells. [file 12896_2021_718_MOESM3_ESM.pdf]
